# Relationship between alcohol intake based on daily smartphone-reported consumption and PEth concentrations in healthy volunteers

**DOI:** 10.1093/alcalc/agae040

**Published:** 2024-06-17

**Authors:** Trine Finanger, Katrine Melby, Olav Spigset, Trine N Andreassen, Stian Lydersen, Ragnhild Bergene Skråstad

**Affiliations:** Clinic of Substance Use and Addiction Medicine, St. Olav University Hospital, 7030 Trondheim, Norway; Department of Clinical and Molecular Medicine, Norwegian University of Science and Technology, 7030 Trondheim, Norway; Clinic of Blue Cross Lade Addiction Treatment Centre, 7041 Trondheim, Norway; Department of Clinical Pharmacology, St. Olav University Hospital, Trondheim, 7030 Norway; Department of Clinical and Molecular Medicine, Norwegian University of Science and Technology, 7030 Trondheim, Norway; Department of Clinical Pharmacology, St. Olav University Hospital, Trondheim, 7030 Norway; Department of Clinical Pharmacology, St. Olav University Hospital, Trondheim, 7030 Norway; Department of Mental Health, Norwegian University of Science and Technology, Trondheim, 7030 Norway; Department of Clinical and Molecular Medicine, Norwegian University of Science and Technology, 7030 Trondheim, Norway; Department of Clinical Pharmacology, St. Olav University Hospital, Trondheim, 7030 Norway

**Keywords:** phosphatidylethanol, digital recording, PEth, TLFB, Timeline Followback, alcohol

## Abstract

**Aims:**

To investigate the association between alcohol consumption registered daily with a digital smartphone-based diary and concentration of phosphatidylethanol (PEth) 16:0/18:1 in a population without a known alcohol use disorder (AUD), and evaluate whether prospective registration of alcohol consumption is better than retrospective registration and if the association between alcohol intake and PEth was affected by sex or body mass index (BMI).

**Methods:**

A total of 41 women and 21 men without AUD-diagnosis registered their alcohol consumption prospectively with a digital diary for 14 days, and retrospectively with the Timeline Followback method in the same time interval. PEth was measured before and after the registration period.

**Results:**

The correlation between alcohol consumption and PEth varied from 0.65 to 0.87. It did not depend significantly on the reporting method, and was not influenced by sex or BMI. Based on the regression coefficient, a reduction of alcohol consumption by two alcohol units (26 g of pure ethanol) per day would lead to a reduction of the PEth concentration of about 0.1 μmol/l, and vice versa.

**Conclusions:**

There was a good correlation between PEth concentration and alcohol consumption, both when alcohol consumption was reported prospectively and retrospectively. The preferred cut-off for PEth should be adjusted to the level of alcohol consumption considered harmful and a purposeful trade-off between sensitivity and specificity. In order to identify persons with a daily alcohol consumption of more than two or three units of alcohol with a sensitivity of 80% or 90%, we suggest a cut-off of around 0.1 μmol/l.

## Teaser

**Short Summary:** There was a good correlation between alcohol consumption and phosphatidylethanol, which was not significantly different depending on the reporting method and not influenced by sex or body mass index. To identify persons with a daily alcohol consumption of more than two to three units, we suggest a cut-off of around 0.1 μmol/l.

## Introduction

Diagnosing and intervening at an early stage is a key factor in improving the prognostic outcome in patients with alcohol use disorder (AUD) (Skinner and Holt 1983). However, in clinical settings subjects with a harmful use of alcohol or undiagnosed AUD may underreport or deny alcohol intake in fear of sanctions and social stigma (Hoonpongsimanont et al. 2021; Johnston 2022), resulting in delayed diagnosis and intervention. In these situations, use of biomarkers as objective methods to estimate alcohol intake is useful. Phosphatidylethanol (PEth) is one of the most sensitive and specific biomarkers for alcohol intake available today. PEth is a collective term for abnormal phospholipids formed from fatty acids in the membranes of various cell types including red blood cells, in the presence of ethanol (Alling et al. 1983; Gustavsson and Alling 1987). Almost fifty different PEth homologs have been identified, with 1-palmitoyl-2-oleoyl-sn-glycerol-3-phosphoethanol (PEth 16:0/18:1) as one of the most abundant (Helander and Zheng 2009; Gnann et al. 2010; Gnann et al. 2014). It was recently reaffirmed that the 16:0/18:1 homolog is the dominant type and is displaying a high correlation with total PEth indicating its efficiency as a biomarker for measuring PEth (Cameron et al. 2023). Because PEth is formed exclusively in the presence of ethanol and the PEth concentration correlates with the level of alcohol consumption, PEth in blood has been increasingly used as a marker for alcohol intake, both in medical and legal settings (Andresen-Streichert et al. 2018; Selim et al. 2022). Its long detection time and high sensitivity for recent heavy drinking make its application potential broader than for other ethanol biomarkers, although its inter-individual biological variation complicates interpretation (Helander et al. 2019a).

Previous studies, both including healthy volunteers and subjects in treatment for AUD, have evaluated the relationship between PEth and level of alcohol consumption, either to defined quantities of alcohol ingested in experimental settings (Kechagias et al. 2015; Hill-Kapturczak et al. 2018) or to retrospectively register self-reported alcohol intake (Schröck et al. 2017; Helander et al. 2019b; Finanger et al. 2022; Skråstad et al. 2023). One of several available tools for reporting alcohol consumption is the Timeline Followback (TLFB) method. TLFB is a validated, retrospective assessment method where daily alcohol intake in a defined period of time is recalled and registered (Sobell and Sobell 1992). TLFB has shown a good correspondence to actual intake in some studies (Sobell et al. 1996; Simpson et al. 2011), while an under-reporting of intake has been noted in others (Toll et al. 2006; Hämäläinen et al. 2020). A possible explanation for this discrepancy could be varying degrees of recall bias. To minimize the risk of such bias, studies where alcohol consumption is recorded continuously are desirable. Helander and colleagues carried out such a study in a population of high consumers (Helander et al. 2019b), but there is a need for similar studies in populations with a presumably lower level of alcohol consumption.

Daily registration of alcohol intake with the use of digital devices, such as smartphones, has shown to be a promising alternative method to TLFB or to prospective registration on paper, and due to the widespread use of digital devices, it is likely to be the preferred tool in the future (Dulin et al. 2017; Recio-Rodriguez et al. 2019). The primary aim of the present study was to investigate the association between the degree of alcohol consumption registered daily with a digital smartphone-based diary and the PEth concentration in blood in a population without known AUD. As we also wanted to explore the extent of the assumed recall bias when reporting alcohol intake, a secondary aim was to study the association between retrospective registration of alcohol consumption by means of TLFB and PEth concentrations, and thereafter to evaluate whether there was a difference between the two registration methods. As the influence on PEth of sex and body mass index (BMI) is not yet fully understood (Hahn et al. 2016; Perilli et al. 2023), the third aim of the study was to examine whether the association between alcohol intake and PEth was affected by sex or BMI.

## Materials and methods

### Participants and study visits

Participants were healthy volunteers recruited from adjacent health and educational institutions. The study period for each participant was 15 days, and the inclusion period was from April to November 2022. Eligible participants were ≥18 years old, did not abstain from alcohol, were self-reported healthy and were able to understand and sign the informed consent form. We advertised for people who usually had an average consumption of ten units of alcohol or more a week. Inclusion was also timed to periods when a significant alcohol intake could be expected to be maintained (Easter, autumn student festival, Christmas party season). We attempted to avoid including people who were abstinent during the period, as this would make it difficult to explore the study’s aim. However, we emphasized that the study participants should not drink more just because they were part of the study, but that they should maintain their usual drinking pattern.

**Figure 1:**
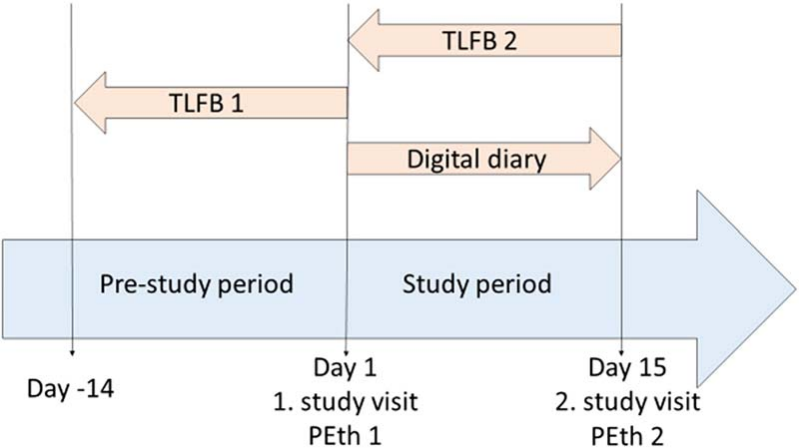
Schematic illustration of Study 1 design. TLFB = Alcohol consumption registered by Timeline Followback; Digital diary = Alcohol consumption registered by digital diary; PEth = Measurement of phosphatidylethanol concentration in blood.

The study design is illustrated in Figure 1. On the inclusion day (Day 1), participants signed the consent form, and sex, age, occupation, body weight and height were recorded. The TLFB method was explained as well as the use of the study-specific digital diary for daily alcohol intake registration. On the same day the participants completed a TLFB form for the past 14 days, pre-study period, (TLFB 1), and blood for PEth analysis was drawn on EDTA (*ethylenediaminetetraacetic acid)* tubes from the cubital vein (PEth 1). During the next 14 days, study period, (Days 1–14) the participants recorded their alcohol consumption prospectively on a daily basis using the digital diary. On Day 15, participants returned to the study site to complete a TLFB form for the past 14 days (TLFB 2). Moreover, a second PEth blood sample was obtained (PEth 2). The participants were informed about the importance of not having ethanol in the blood at the time of blood sampling to avoid in vitro formation of PEth, but a breathalyzer or blood alcohol concentration test was not performed.

### Digital diary

Data in the study were collected using a specifically developed tool, the eFORSK tool, based on an independent digital solution for electronic data collection in clinical studies owned and operated by HEMIT (Helse Midt-Norge IT, i.e. the IT organization affiliated to the Central Norway Regional Health Authority). eFORSK follows Norwegian regulations for privacy and secure transmission and storage of health research data.

Upon inclusion, the participants filled in a digital informed consent form and a form with anamnestic data. The participants received a daily reminder on their smartphone to report daily alcohol consumption in a form with drop-down menus of different types of alcoholic beverages (hereafter called “digital diary”). A study day in the digital diary was from 12.00 pm (noon) to 11.59 am the next day. This day shift adjustment was made because drinking sessions tend to extend past midnight, and a division in date at midnight would therefore be unnatural. Participants did not have the opportunity to go back to check or adjust their answers in the digital diary.

### Timeline Followback

TLFB is a retrospective drinking assessment method where daily alcohol intake over a specified time period is recorded on a calendar for up to 12 months prior to the interview date (Sobell 1992). The method is validated and well known in clinical and research use to estimate both the alcohol intake and number of drinking days (Supplementary file 1). TLFB has shown good correspondence to actual alcohol intake in some studies (Sobell et al. 1996; Simpson et al. 2011), whereas under-reporting has been noted in others (Toll et al. 2006; Hämäläinen et al. 2020). Under-reporting has mainly been observed in patients suffering from harmful alcohol use or dependence, while agreement has been good in groups without a drinking problem (Boniface et al. 2014). The participants were asked to recall the number of alcohol units consumed per day during the last 14 days prior to the study visits at Days 1 and 15.

### Alcohol units

There is no international consensus on the definition of how much pure alcohol a standard drink contains and it varies between 8 g and 20 g between different countries (Wikipedia 2024). In this study, one alcohol unit would contain ~13 g of ethanol, which is the most common definition of the content in an alcohol unit used in Norway. Both the TLFB and the digital diary recording used the following definitions of one alcohol unit: .33 l of beer (4.5%), one glass (0.125 l, 12%) of wine, one drink (0.04 l, 40%) of spirit. One bottle of wine was calculated to contain six glasses of wine.

### Analysis of PEth

Blood was stored at −80°C from sampling until analysis. The PEth analog 16:0/18:1 was analyzed based on a validated routine ultra-performance liquid chromatography–tandem mass spectrometry (UPLC®–MSMS) method with a quantification range of 0.030–4.0 μmol/l (use a factor of 703 to convert to ng/ml), as previously described (Andreassen et al. 2018). For this study, the extraction method was modified to lower the limit of quantification to 0.003 μmol/l, and values below this limit were set to 0.000 μmol/l. The extraction modifications were as follows: the sample volume was increased from 150 μl to 300 μl blood; the total protein precipitation solvent (2-propanol) with the internal standard (PEth 16:0/18:1-d_5_, 0.13 μmol/l) was increased from 450 μl to 1050 μl; and an evaporation (50°C, 60 minutes under stream of air) and resolving (2-propanol, 100 μl) step was added to the sample preparation.

Chromatographic separation was achieved with a Waters Acquity BEH-Phenyl column (2.1 × 30 mm, 1.7 μm) with guard column using a gradient elution starting with 40% ammonium formate (5 mM, pH 10.1, mobile phase A) in combination with 60% acetonitrile (mobile phase B). During the first 90 seconds, the gradient went from 60% to 95% B, it then remained at 95% B for 6 seconds. Forty-two seconds with 60% B was found sufficient to equilibrate the column before next injection. The flow rate was .5 ml/min and the total run time was 2.3 minutes. The injection volume was 2 μl. For detection and quantification of PEth 16:0/18:1 in negative ionization mode, the *m*/*z* 701.7 > 255.2 and *m*/*z* 701.7 > 281.3 transitions were used. For the internal standard PEth 16:0/18:1-d_5_ the *m*/*z* 706.7 > 255.2 transition was used. The five-point calibration curve went from 0.003 μmol/l to 0.50 μmol/l with a quadratic curve fit and a coefficient of determination, *R*^2^, of >0.9999. Coefficients of variation (CV) for between-assay precision (*n* = 5) were ≤7.4% with inaccuracies ≤2.4% at the 0.008 (quality control [QC] 1), 0.04 (QC 2), and 0.4 (QC 3) μmol/l levels. The validation parameters regarding limit of quantification (LOQ), curve fitting, reproducibility (inter-assay precision and accuracy), retention time, and ion ratio were revalidated after the change. Consequently, all validation parameters that could possibly have been affected by the method change were revalidated.

The PEth concentration is reported in μmol/l. For the conversion between μmol/l and ng/ml (or μg/l) use a conversion factor of 703 (Helander and Hansson 2023).

### Statistics

We used linear regression with PEth as dependent variable and average number of alcohol units per day as covariate, unadjusted and adjusted for age and sex. This was done separately for the two measures of self-reported alcohol consumption. Next, to evaluate whether sex and BMI moderated these associations we also included sex and the interaction between sex and alcohol units in the unadjusted analyses, and similarly for BMI. The Pearson’s correlation coefficient between PEth and each of the two measures of self-reported alcohol consumption was calculated. The two correlation coefficients were compared using the significance test recommended by Meng et al. (1992).

We constructed receiver operating characteristics (ROC) curves for PEth concentration as predictor for digital diary-reported alcohol consumption of more than one, two, three, and four alcohol units per day, respectively. We identified the PEth concentrations which gave sensitivities of at least 80% and 90%, respectively, with corresponding specificities.

We report 95% confidence intervals (CI) when relevant, and regarded two-sided *P*-values below .05 to represent statistical significance. Analyses were carried out using Microsoft Excel and SPSS 29.

### Ethics

The study was approved by The Regional Committee for Medical and Health Research Ethics in Central Norway (2019/511). The study was conducted in accordance to the principles laid down in the Declaration of Helsinki (World Medical Association 2013). All participants gave their informed consent before inclusion in the study. Data were handled in accordance with the EU General Data Protection Regulation (GDPR). The participants received financial compensation of NOK 500 (~45 EUR or 45 USD) for lost income after completing the study.

## Results

None of the 62 participants included in the study were lost to follow-up. One participant was unable to complete the digital diary registration (the required two-factor authentication failed), but finished the registration on paper. For this person we also lacked information on body weight and height. Data from this person were included only in analyses where these data were needed.

The mean (SD) age of the participants was 29.8 (10.1) years and 41 (66%) were women. Participant characteristics, PEth concentrations and reported alcohol consumption are presented in Table 1.

**Table 1:** Participant characteristics, PEth concentrations and self-reported alcohol consumption in mean number of alcohol units per day. No data were missing except for one male participant with no information about digital diary registration and BMI.

	***Entire group***	***Women***	***Men***
	N = 62	N = 41	N = 21
	Mean (SD) min - max	Mean (SD) min - max	Mean (SD) min - max
Age, years	29.8 (1.1) 20–57	29.9 (10.3) 20–57	29.7 (1.0) 21–49
BMI, kg/m^2^ (n = 61)	24.4 (3.7) 17.0–39.0	24.2 (3.6) 18.8–39.0	24.8 (3.9) 17.0–32.9
Occupation n (%)			
Student	37 (60%)	24 (59%)	13 (62%)
Employee	25 (40%)	17 (41%)	8 (38%)
PEth 1 concentration, μmol/l	0.139 (.148) .000–.751	0.117 (.134) .000–.751	0.183 (.166) .000–.672
PEth 2 concentration, μmol/l	0.114 (.100) .000–.487	0.099 (.094) .000–.399	0.143 (.107) .020–.487
Alcohol units per day, TLFB 1	2.1 (1.6) .0–6.8	1.7 (1.3) .1–6.8	2.9 (1.8) .0–6.6
Alcohol units per day, digital diary	2.3 (1.6) .0–8.3	1.9 (1.5) .0–8.3	3.1 (1.5) .6–6.9
Alcohol units per day, TLFB 2	2.0 (1.4) .0–7.1	1.6 (1.3) .0–6.7	2.7 (1.5) .4–7.1

BMI: Body mass index, TLFB: Timeline Followback

According to the digital diary registration, total alcohol consumption during the 14 study days ranged from 0 to 116.5 alcohol units, with a mean of 32.1 (SD 22.2). The individual daily alcohol consumption varied from 0 to 26 alcohol units. Based on the digital diary registration, a total of 47 (76%) of the participants on average consumed more than one alcohol unit per day, 35 (56%) consumed more than two alcohol units per day, 15 (24%) consumed more than three alcohol units per day and 6 (10%) consumed more than four alcohol units per day.

PEth concentrations at inclusion (PEth 1) and on day 15 (PEth 2) are presented in Table 1. At inclusion four participants had a PEth concentration (PEth 1) below the limit of detection (0.003 μmol/l). A total of 12 participants (19%) had a PEth 1 concentration below 0.030 μmol/l, while five participants (8%) had a PEth 1 value at or above 0.300 μmol/l. After the 14 days registration period (PEth 2), only one participant had a concentration below the limit of detection (0.003 μmol/l). This participant had a PEth 1 concentration below the limit of detection, and thus never had a detectable PEth concentration in blood. The same participant reported consuming a total of 2.5 alcohol units during the 14-day study period (digital diary), and two alcohol units during the two last weeks prior to inclusion (TLFB 1). A total of 14 persons (23%) had a PEth 2 concentration below 0.030 μmol/l, and among these, the average number of alcohol units consumed per day in the last 14 days (digital diary) was 0.6 (range 0–1.7). In the subgroup of 45 persons (73%) with a PEth two concentration between 0.030 and 0.299, the average alcohol consumption in the study period (digital diary) was 2.5 units per day (range 0.7–5.3). Among the three persons (5%) with a PEth two concentration at or above 0.30 μmol/l the average alcohol consumption was 6.4 units per day (range 3.9–8.3).

**Figure 2:**
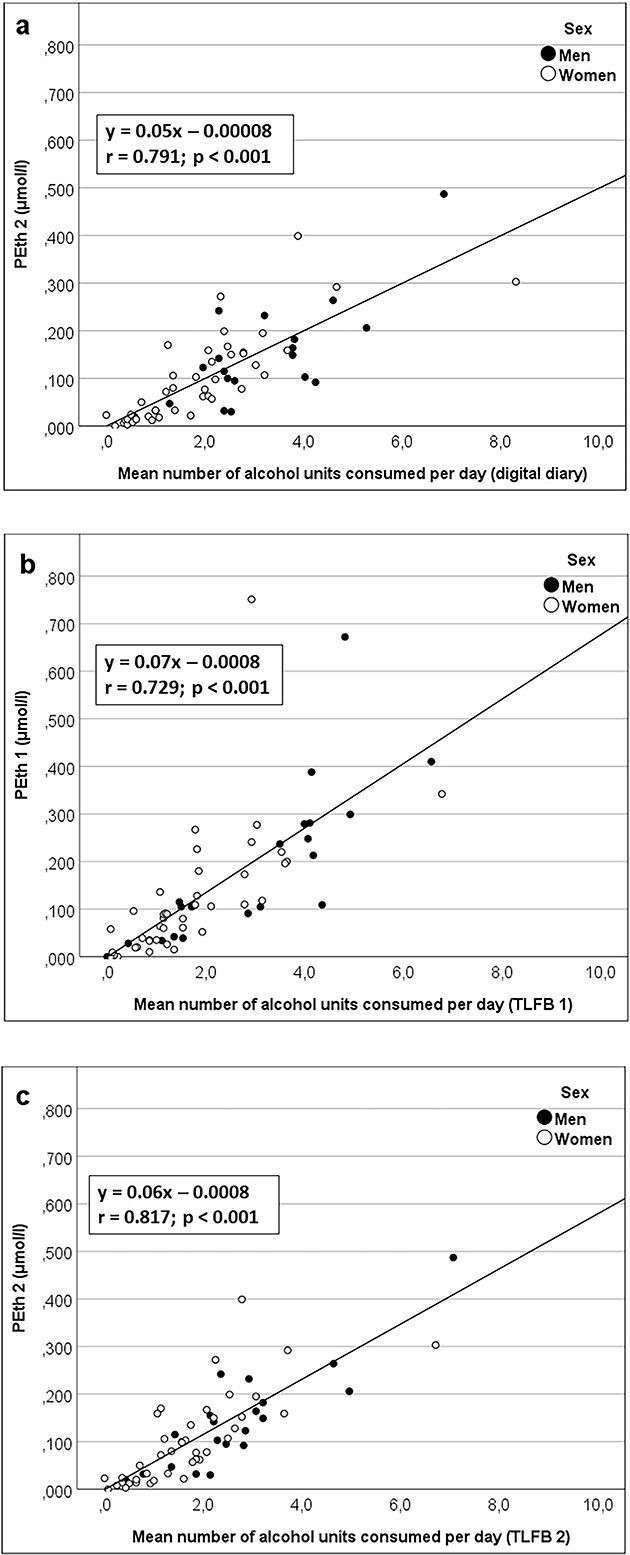
Scatter plots illustrating the relationship between (a) alcohol consumption recorded with digital diary after 14 days and PEth concentration after 14 days (PEth 2), (b) Alcohol consumption recorded with TLFB at inclusion (TLFB 1) and PEth concentration at inclusion (PEth1) and (c) Alcohol consumption recorded with TLFB after 14 days (TLFB 2) and PEth concentration after 14 days (PEth).

The relationship between self-reported alcohol consumption and PEth concentrations is illustrated in Figure 2, Supplementary Figures SS1 and SS2. The estimated regression coefficient for the relationship between mean reported alcohol consumption (digital diary) and PEth 2 was 0.050 (CI = 0.040–0.060) The results were substantially the same when adjusting for age and sex, as this adjustment changed the regression coefficient to 0.047 (CI = 0.036–0.057). Including interactions with sex or BMI gave no significant interaction effects. The Pearson correlation coefficients ranged from 0.65 to 0.87, both when alcohol consumption was reported with digital diary, TLFB 1 and TLFB 2, this applied in the whole group as well as among women and men separately (Supplementary Table SS1).

There was a high correlation between mean number of alcohol units consumed per day reported with digital diary and with TLFB after 14 days (TLFB 2) both for the entire group (*r* = 0.96; CI = 0.94–0.98) and for women (*r* = 0.98; CI = 0.96–0.99) and men (*r* = 0.92; CI = 0.82–0.97) separately. There were no significant differences between the correlation coefficients for digital diary registration and PEth 2 concentration and the correlation coefficients between registration by TLFB2 and PEth2 concentration, neither for the entire group (*P* = 0.21) nor for women (*P* = 0.27). However, the difference between the correlation coefficients (*r* = 0.77 for digital diary vs. PEth and *r* = 0.87 for TLFB2 vs. PEth) was marginally statistically significant for men (*P* = 0.042).

**Figure 3:**
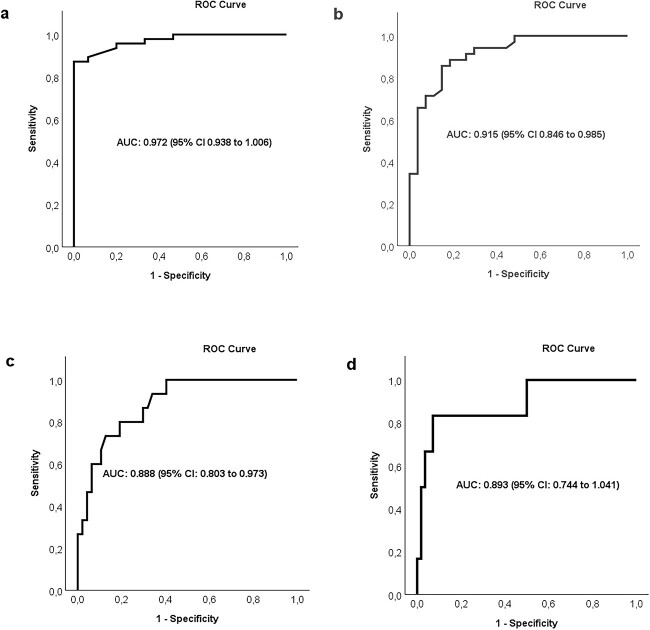
ROC curves for PEth 2 identifying participants consuming (a) more than 1 alcohol unit per day (*N* = 47), (b) more than 2 alcohol units per day (*N* = 35), (c) more than 3 alcohol units per day (*N* = 15), and (d) more than four units per day (*N* = 6) according to the digital diary

The ability of PEth to detect individuals having a mean intake of more than one, two, three and four alcohol units per day reported by the digital diary, were evaluated with ROC curves (Fig. 3). Specificities at fixed 90% and 80% sensitivities are presented in Table 2 and Supplementary Table SS2.

**Table 2:** Cut-off concentrations for PEth and corresponding specificities to obtain at least 90% or 80% sensitivities to classify drinking above 1, 2, 3 or 4 alcohol units per day, based on data shown in Supplementary Table S2.

	**PEth cut-off concentration (μmol/l)**	**Specificity (%)**
**Identification of persons drinking > 1 alcohol unit/day**		
90% sensitivity	0.033	86.7
80% sensitivity	0.068	100
**Identification of persons drinking > 2 alcohol units/day**		
90% sensitivity	0.063	74.1
80% sensitivity	0.097	85.2
**Identification of persons drinking > 3 alcohol units/day**		
90% sensitivity	0.102	66.0
80% sensitivity	0.146	80.9
**Identification of persons drinking > 4 alcohol units/day**		
90% sensitivity^a^	-	-
80% sensitivity	0.203	92.9

a Not calculated due to too few participants included

## Discussion

The primary aim of the present study was to investigate the association between alcohol consumption registered on a daily basis with a digital smartphone-based diary and the PEth concentration in blood. Theoretically, this registration method should be close to a gold standard to quantify alcohol consumption, which is important since the medical risk associated with alcohol use increases with alcohol intake. Moreover, to minimize the risk of under-reporting all study participants were healthy volunteers with no known AUD. Thus, the chosen design should have been optimized for exploring the relationship between alcohol consumption and PEth concentrations. The main finding of a strong correlation between self-reported alcohol consumption and PEth concentrations, both when alcohol consumption was reported with a digital diary and when it was reported with TLFB, confirms this assumption. Thus, we consider that most of the demonstrated variability between alcohol intake and PEth concentrations in the present study could be caused by inter-individual biological factors rather than by reporting irregularities.

Based on the regression line in Figure 2a, a reduction of alcohol consumption of two alcohol units (26 g of pure ethanol) per day in an individual would lead to a reduction of the PEth concentration of about .10 μmol/l and vice versa. This result is in line with the finding in another study, where an increase in consumption of ~20 g of ethanol per day would increase the PEth value by ~0.10 μmol/l (Helander et al. 2019b).

There is no unambiguous international consensus on how a given PEth concentration should be interpreted in terms of alcohol consumption. Studies have suggested varying cut-off values for PEth, detecting different levels of alcohol consumption with different levels of sensitivities and specificities (Afshar et al. 2017; Jørgenrud et al. 2021; Hasken et al. 2023). However, it is widespread to interpret the concentrations in line with the guidelines presented in a recent report from the Society of PEth Research (PEth-NET) (Luginbühl et al. 2022). According to these, PEth concentrations below 0.03 μmol/l are compatible with abstinence or very low alcohol consumption, whereas concentrations at or above 0.3 μmol/l are consistent with chronic excessive alcohol consumption, defined as an average consumption of 60 g or more for men and 40 g or more for women of pure ethanol on a single drinking day over a prolonged duration. By applying the definition of one alcohol unit (~13 g) from the present study, these limits correspond to a consumption of at least 4.6 units for men and 3.1 units for women. In our study, the median PEth value in those who had an intake of between 3.1 and 4 units per day was 0.164 μmol/l. Among the six people with an average daily alcohol consumption more than 4 units, the median PEth value was 0.278 μmol/l. Thus, these values are lower than 0.3 μmol/l concentration suggested by the PEth-NET group (Luginbühl et al. 2022).

Furthermore, ROC analyses from our study showed that for detecting an intake of more than three alcohol units per day with sensitivities of 90% and 80%, cut-off concentrations of 0.102 μmol/l and 0.146 μmol/l, respectively, should be applied. To detect an intake of more than four units per day, the cut-off concentration with 80% sensitivity was 0.203 μmol/l. Of the total of 15 people who reported drinking more than three units per day, only three had a PEth value above 0.3 μmol/l, giving a sensitivity of only 20% when applying a cut-off concentration of 0.3 μmol/l (corresponding specificity 100%).

In a previously published study from our group, we examined the relationship between PEth concentrations and self-reported alcohol consumption in 24 574 individuals included in the Norwegian HUNT-study (Skråstad et al. 2023). In both that study and the present, the proposed cut-offs for detection of alcohol consumption in the order of magnitude of 3 units per day were below 0.150 μmol/l. This supports the conclusion that if it is desired to detect an alcohol consumption above approximately 3 units per day with a high sensitivity, the cut-off should probably be lower than 0.3 μmol/l and perhaps as low as 0.10 μmol/l. In contrast, if an approximately 100% specificity is required, the 0.3 μmol/l cut-off is suitable.

The use of a digital diary recording of alcohol consumption did not show better correlation with PEth concentrations than the retrospective TLFB questionnaire. These findings are not in line with the results of other studies (Dulin et al. 2017; Merrill et al. 2020). One explanation could be that the daily digital recording made the study participants more aware of their alcohol consumption and that it therefore was easier for them to remember the correct amount of alcohol consumed when they completed the TLFB after the registration period of 2 weeks. Another explanation could be that the study population consisted of healthy volunteers without AUD with regular lives as employees or students, and no advantages or sanctions related to under- or over-reporting. Together, these factors probably optimize the conditions for a correct recall, which suggests that the TLFB is a well-suited tool for reporting alcohol intake in similar populations.

Our study did not demonstrate any influence of sex or BMI on the correlation between alcohol consumption and PEth, which is in line with the findings in previous studies (Helander et al. 2019a; Hahn et al. 2021). This finding is in contrast to the influence of sex on the relationship between amount of ethanol consumed and the blood alcohol concentration (Norberg et al. 2003). However, investigating the effect of sex was not a primary aim of the study, and the sample size might not have been sufficient to identify such effects. The same might apply to the lack of any effect of BMI, as an impact of BMI has been observed in some other studies (Hahn et al. 2021). Another reason why we did not observe an effect of BMI in our material could be that it is total body water rather than BMI that primarily affects the relationship between the amount of ethanol consumed and blood alcohol concentration, and thus also PEth (Jones 2019). Unfortunately, we did not have a measure of body composition in the present study. In addition, it may be that our study was not sufficiently large to demonstrate an effect of BMI, particularly as we had few participants with very low and/or very high BMI. A suggestion for future research would be to design a larger study with sufficiently detailed measures of body composition.

The present study has some strengths and weaknesses that should be addressed. A main strength is that the participants recorded their alcohol consumption prospectively on a daily basis and that the digital solution for registration provided participants with daily reminders to register intake. However, due to the national legislation regarding transfer and storage of research data, the study participants had to use two-factor authentication every time they registered their data. This made the registration more impractical, and some participants therefore ended up registering data a few days late. Thus, registration could have been less accurate in these cases. Another possible weakness is that the study participants may still have misreported because they quickly forgot the amount of alcohol consumed or estimated the amount incorrectly. Although participants have accurately reported the fluid volume consumed precisely for each of the beer, wine and spirits classes, there is an inherent uncertainty related to the actual amount of alcohol consumed. For example, a white wine with 7% alcohol by volume (ABV) contains just half as much alcohol as a red wine with 14% ABV, even though both are classified as “wine”. Despite these limitations, we consider that the method used to record alcohol consumption is as accurate and traceable as possible, given the current regulations we had to adhere to. If consumption is to be determined more precisely, it must be done in an experimental setting where the study participants consume a given amount of alcohol in grams.

We assumed that the participants were sober, as blood samples were taken between lectures at the university or during working hours. This was not confirmed with a breathalyzer or blood alcohol concentration test, and could potentially lead to PEth formation after sampling. In addition, the results from this study do not take into account the drinking pattern and how it could affect the PEth concentration. In our analysis only the average number of alcohol units consumed per day was used, but this number varied a lot from day to day. It is reasonable to believe that in participants where most of the alcohol intake took place close to the time for blood sampling, the PEth concentration will be higher than when most of the intake took place early in the registration period.

## Conclusion

The main finding of this study is that there was a high correlation between self-reported alcohol consumption and the PEth concentration in healthy volunteers. The correlations were not significantly different between prospective and retrospective registration. Moreover sex, BMI, and age did not seem to affect the PEth concentrations. The preferred PEth cut-off for defining suspected harmful use in a population must be related to which level of alcohol consumption that is considered detrimental, and also whether a high sensitivity or a high specificity is preferred. In order to identify persons with a daily alcohol consumption of more than two or three units of alcohol with a sensitivity of 80% or 90%, we suggest a cut-off of about 0.1 μmol/l.

## Supplementary Material

Supplementary_figure_1_illustration_agae040

Supplementary_figure_2_illustration_agae040

Supplementary_table_1_revised_agae040

Supplementary_table_2_agae040

Supplementary_figure_legends_agae040

## Data Availability

The authors confirm that the data supporting the findings of this study are available within the article and its supplementary materials and can be made available from the corresponding author upon reasonable request.

## Notes

Note: The conversion factor for PEth from μmol/l to ng/ml (or μg/l) is 703.
