# Supplementary figures and images for: Relationship between alcohol intake based on daily smartphone-reported consumption and PEth concentrations in healthy volunteers
Source: Alcohol Alcohol. 2024 Jun 17;59(4):agae040. doi: 10.1093/alcalc/agae040 (PMC11180986; doi:10.1093/alcalc/agae040)

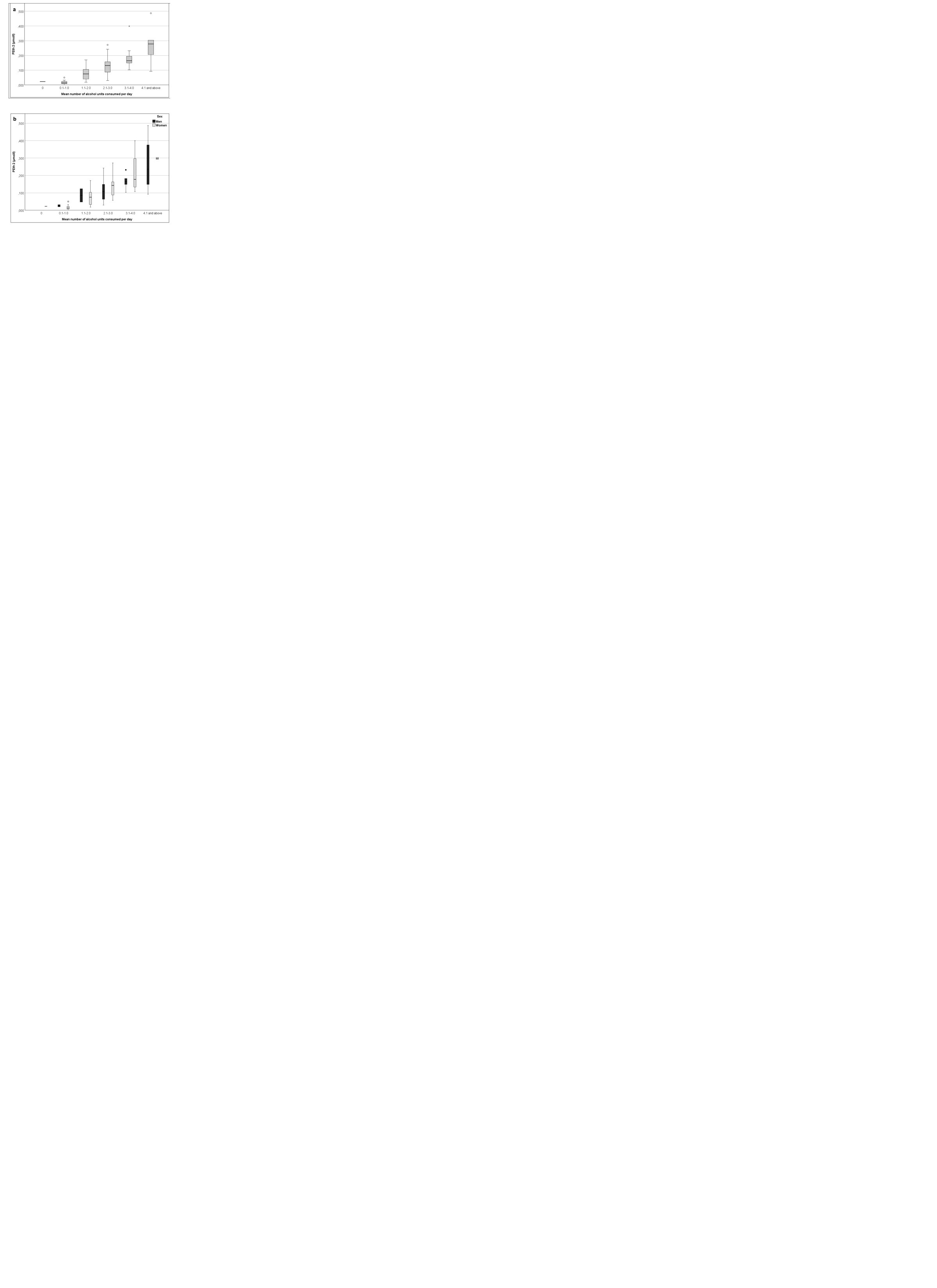

Supplement: Supplementary_figure_1_illustration_agae040 [file supplementary_figure_1_illustration_agae040.jpeg]

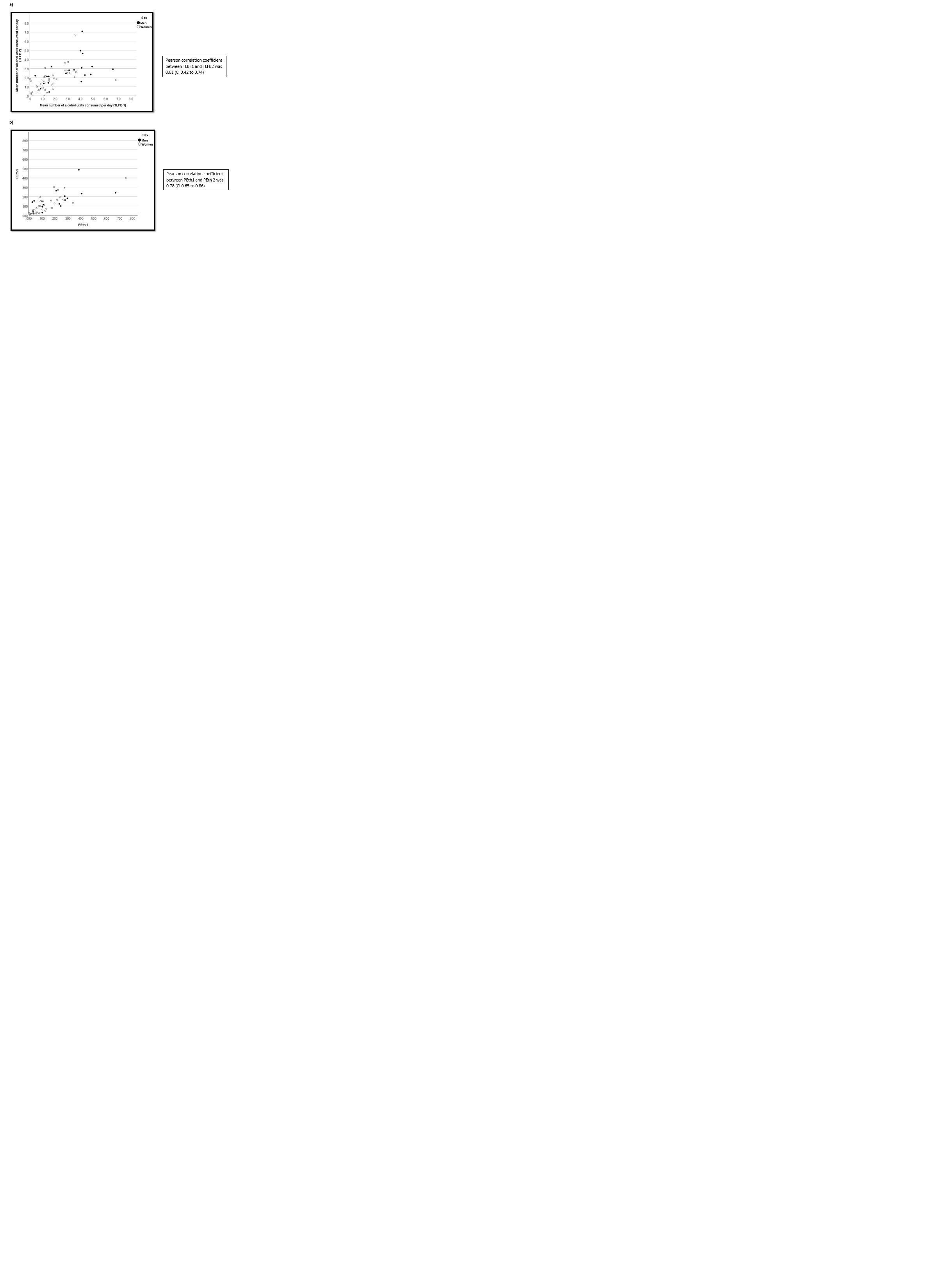

Supplement: Supplementary_figure_2_illustration_agae040 [file supplementary_figure_2_illustration_agae040.jpeg]
