# Supplementary material for: Relationship between alcohol intake based on daily smartphone-reported consumption and PEth concentrations in healthy volunteers
Source: Alcohol Alcohol. 2024 Jun 17;59(4):agae040. doi: 10.1093/alcalc/agae040 (PMC11180986; doi:10.1093/alcalc/agae040)
Supplement: Supplementary_table_1_revised_agae040 [file supplementary_table_1_revised_agae040.docx]

**Supplementary table 1** Pearson correlation coefficients for i) mean number of alcohol units consumed per day reported with Timeline Followback at inclusion (TLFB 1) and PEth concentration at inclusion (PEth 1), ii) mean number of alcohol units consumed per day reported with digital diary and PEth concentration after 14 days (PEth 2) and iii) mean number of alcohol units consumed per day reported with Timeline Followback after 14 days (TLFB 2) and PEth concentration after 14 days (PEth 2). Estimates and 95% Cis.

|  | ***Entire group*** | ***Women*** | ***Men*** |
| --- | --- | --- | --- |
| Digital diary vs. PEth 2 | 0.79 (0.67 to 0.87) | 0.79 (0.64 to 0.88) | 0.78 (0.52 to 0.90) |
| TLFB 1 vs. PEth 1 | 0.73 (0.59 to 0.83) | 0.65 (0.43 to 0.80) | 0.80 (0.57 to 0.92) |
| TLFB 2 vs. PEth 2 | 0.82 (0.71 to 0.89) | 0.77 (0.61 to 0.87) | 0.87 (0.71 to 0.95) |
