# Supplementary material for: Relationship between alcohol intake based on daily smartphone-reported consumption and PEth concentrations in healthy volunteers
Source: Alcohol Alcohol. 2024 Jun 17;59(4):agae040. doi: 10.1093/alcalc/agae040 (PMC11180986; doi:10.1093/alcalc/agae040)
Supplement: Supplementary_table_2_agae040 [file supplementary_table_2_agae040.docx]

**Supplementary table 2** Cross-tables underlying the results displayed in Table 2.

1. At least 90% sensitivity for detection of consumption of more than 1 alcohol unit per day

|  | **PEth ≤ 0.033 µmol/l** | **PEth ˃ 0.033 µmol/l** | **Total** |
| --- | --- | --- | --- |
| **≤1 AU/day** | 14 (93.3%) | 1 (6.7%) | 15 |
| **> 1 AU/day** | 5 (10.6%) | 42 (89.4%) | 47 |
| **Total** | 19 | 43 | 62 |

1. At least 80% sensitivity for detection of consumption of more than 1 alcohol unit per day

|  | **PEth ≤ 0.068 µmol/l** | **PEth ˃ 0.068 µmol/l** | **Total** |
| --- | --- | --- | --- |
| **≤1 AU/day** | 15 (100%) | 0 (0%) | 15 |
| **> 1 AU/day** | 9 (19.1%) | 38 (80.9%) | 47 |
| **Total** | 24 | 38 | 62 |

1. At least 90% sensitivity for detection of consumption of more than 2 alcohol units per day

|  | **PEth ≤ 0.063 µmol/l** | **PEth ˃ 0.063 µmol/l** | **Total** |
| --- | --- | --- | --- |
| **≤2 AU/day** | 20 (74.1%) | 7 (25.9%) | 27 |
| **> 2 AU/day** | 4 (11.4%) | 31 (88.6%) | 35 |
| **Total** | 24 | 38 | 62 |

1. At least 80% sensitivity for detection of consumption of more than 2 alcohol units per day

|  | **PEth ≤ 0.097 µmol/l** | **PEth ˃ 0.097 µmol/l** | **Total** |
| --- | --- | --- | --- |
| **≤2 AU/day** | 23 (85.2%) | 4 (14.8%) | 27 |
| **> 2 AU/day** | 7 (20.0%) | 28 (80.0%) | 35 |
| **Total** | 30 | 32 | 62 |

1. At least 90% sensitivity for detection of consumption of more than 3 alcohol units per day

|  | **PEth ≤ 0.102 µmol/l** | **PEth ˃ 0.102 µmol/l** | **Total** |
| --- | --- | --- | --- |
| **≤3 AU/day** | 31 (66.0%) | 16 (34.0%) | 47 |
| **> 3 AU/day** | 1 (6.7%) | 14 (93.3%) | 15 |
| **Total** | 32 | 30 | 62 |

1. At least 80% sensitivity for detection of consumption of more than 3 alcohol unit per day

|  | **PEth ≤0.146 µmol/l** | **PEth ˃ 0.146 µmol/l** | **Total** |
| --- | --- | --- | --- |
| **≤3 AU/day** | 38 (80.9%) | 9 (19.1%) | 47 |
| **> 3 AU/day** | 3 (20.0%) | 12 (80.0%) | 15 |
| **Total** | 41 | 21 | 62 |

1. At least 80% sensitivity for detection of consumption of more than 4 alcohol units per day

|  | **PEth 0.203 ≤ µmol/l** | **PEth ˃ 0.203 µmol/l** | **Total** |
| --- | --- | --- | --- |
| **≤4 AU/day** | 52 (92.9%) | 4 (7.1%) | 56 |
| **> 4 AU/day** | 1(16.7%) | 5 (83.3%) | 6 |
| **Total** | 53 | 9 | 62 |
