# Supplementary material for: Relationship between alcohol intake based on daily smartphone-reported consumption and PEth concentrations in healthy volunteers
Source: Alcohol Alcohol. 2024 Jun 17;59(4):agae040. doi: 10.1093/alcalc/agae040 (PMC11180986; doi:10.1093/alcalc/agae040)
Supplement: Supplementary_figure_legends_agae040 [file supplementary_figure_legends_agae040.docx]

**Supplementary figure 1** Boxplot describing the relationship between alcohol consumption recorded with digital diary after 14 days and PEth concentrations after 14 days (PEth 2) in a) the entire group and b) men and women shown separately. Boxes represent the interval between the first and third quartiles, the horizontal line represents median, whiskers represent minimum and maximum levels excluding outliers. Circles represent outliers with concentrations between 1.5 and 3 times the interquartile range from the median.

**Supplementary figure 2** Scatter plot comparing a) alcohol consumption recorded with TLFB at inclusion (TLFB 1) versus alcohol consumption recorded with TLFB after 14 days (TLFB 2) and b) PEth concentration at inclusion (PEth 1) versus PEth concentration after 14 days (PEth 2).
